# Valproic Acid Concentrations in Mothers, Colostrum and Breastfed Infants during the Early Postpartum Period: Comparison with Concentrations Determined during Delivery and in the Mature Milk Period

**DOI:** 10.3390/pharmaceutics13122074

**Published:** 2021-12-03

**Authors:** Ivana Kacirova, Milan Grundmann, Hana Brozmanova

**Affiliations:** 1 Department of Clinical Pharmacology, Faculty of Medicine, University of Ostrava, 70300 Ostrava, Czech Republic; ivana.kacirova@fno.cz (I.K.); hana.brozmanova@fno.cz (H.B.); 2 Department of Laboratory Medicine, Institute of Clinical Pharmacology, University Hospital Ostrava, 70852 Ostrava, Czech Republic

**Keywords:** valproic acid, colostrum, breastfeeding

## Abstract

To obtain information on the transport of valproic acid from mothers to colostrum and breastfed infants, in this cohort study, valproic acid concentrations in maternal serum (90 subjects), colostrum and the serum of breastfed infants were analyzed in years 1993–2018, between the 2nd and 5th postnatal days. Valproic acid concentrations ranged from 4.3 to 66.5 mg/L (mean 31.2 ± 13.6 mg/L) in maternal serum, from 0.5 to 5.9 mg/L (mean 1.1 ± 1.2 mg/L) in milk, and from 0.5 to 42.9 mg/L (mean 15.4 ± 9.4 mg/L) in infant serum. The milk/maternal serum concentration ratio ranged from 0.01 to 0.22 (mean 0.04 ± 0.04), and the infant/maternal serum concentration ratio ranged from 0.01 to 1.61 (mean 0.51 ± 0.28). A significant correlation was found between serum concentrations of breastfed infants and milk concentrations, maternal serum concentrations, maternal daily dose, and dose related to maternal body weight. Valproic acid concentrations in milk and infant serum did not reach the lower limit of the reference range used for the general epileptic population, and three-quarters of the concentrations in milk were lower than the lower limit of quantification. Routine monitoring of serum concentrations of breastfed infants is not necessary. If signs of potential adverse reactions are noted, serum concentrations of the infants should be measured.

## 1. Introduction

Valproic acid (VPA) has become one of the most widely used antiseizure medications (ASMs) worldwide for the treatment of both generalized and focal seizures. Unfortunately, a major concern for VPA use in women is its teratogenic potential, which increases the risk of major congenital malformations (MCMs) 5.69-fold as compared to children of mothers without epilepsy, 3.13-fold for children of mothers with untreated epilepsy, and significantly relative to fetuses exposed to other ASMs. Prenatal VPA exposure also has negative long-term effects with a higher risk of attention-deficit hyperactivity disorder and worse school performance compared to children exposed to other or no ASM. In 2014, the risks of MCM and cognitive delay associated with intrauterine VPA exposure caused regulatory agencies, the U.S. Food and Drug Administration (FDA) and the European Medicines Agency (EMA), to restrict usage of VPA in women of fertile age [1,2,3,4]. In 2018, the EMA endorsed new measures to avoid exposure of babies to VPA in the womb, which include a ban on the use of such medicines for migraine or bipolar disorder during pregnancy and a ban on using it to treat epilepsy during pregnancy unless there is no other effective treatment available [5]. 

However, only controversial information is available from clinical practice concerning the magnitude of change that restricting VPA use will achieve regarding fetal malformation rates and seizure disorder control during pregnancy in the same population. Cerelli Irelli et al. [6] demonstrated decreased control of generalized epilepsy when VPA therapy was withdrawn during pregnancy. The study of Tomson et al. [7], describing the consequences of changing patterns of ASM use, which included decreased VPA use in pregnant women with epilepsy, found a decreased hazard of fetal malformation without an appreciable deterioration in generalized tonic–clonic seizure control. Data from the Australian Pregnancy Register [8] showed a similar finding in relation to a reduced risk of teratogenicity when pre-pregnancy cessation of VPA or reduction of the dose were applied. In contrast to these substantial benefits, seizure control was worse when VPA was ceased or reduced, and these findings suggest that appreciable numbers of women with generalized seizures and other types of epilepsies will be disadvantaged by impaired control of their seizure disorders [8]. The prescription of VPA to girls/women in general and during pregnancy has decreased significantly in the past decade in many countries [6,7,8,9,10], but older ASMs, including VPA, are still widely used in low- and middle-income countries such as India, the Kingdom of Bhutan, and southern Caribbean countries. The higher cost of newer ASMs, such as lamotrigine and levetiracetam, their limited availability in the public distribution system, and the lack of familiarity with updated guidelines by care providers, are important barriers to their wider use [11,12,13]. 

In 2015, we examined the transmission of VPA through the placenta, and in 2019, we examined the transport of VPA from breastfeeding mothers to mature milk and breastfed infants [14,15]. Because breast milk contains mainly colostrum in the first 2–5 days after birth and the milk is mature only at the end of the first week after birth, the aim of our current study was to analyze the concentrations of VPA in colostrum. Concentrations measured in breastfed infants were compared with paired concentrations in the umbilical cord and with serum concentrations measured in breastfed infants during the mature milk period [14,15]. The effect of comedication with other ASMs on increasing VPA elimination was also analyzed [16].

## 2. Materials and Methods

Inclusion criteria: Data of women with epilepsy treated by VPA during colostrum period were analyzed. Application forms for routine therapeutic drug monitoring (TDM) and serum concentrations of mothers, milk (i.e., colostrum) and breastfed infants collected at our department in the years 1993–2018 were used as a source of data. This study was appropriately reviewed and approved by the local Ethics Committee (Reference number 857/2021, The Ethics Committee of University Hospital Ostrava, Czech Republic). Written informed consent before enrollment in the study was not required for routine TDM. Exclusion criteria: serum and/or milk samples and request forms for routine TDM from other patients which did not fulfil the inclusion criteria. 

This cohort study included information on 90 women (aged 28 ± 5 years) and their 78 breastfed infants (41 girls, 37 boys; one pair of twins) with a weight of 3.2 ± 0.5 kg and a length of 49 ± 2 cm. Breastfeeding mothers have used VPA to treat epilepsy either alone (or in combination with the ‘neutral’ antiseizure medication levetiracetam) or in combination with enzyme-inducing ASMs (carbamazepine, phenytoin, phenobarbital and primidone) and/or lamotrigine or topiramate. Maternal serum, milk (i.e., colostrum) and infant serum samples were collected between the 2nd and 5th postnatal days (median 3 days), usually before the morning maternal dose. For VPA concentration analysis, there was one vial of colostrum taken with a volume of about 4.0 mL. Then, the VPA concentrations were measured in our department. For statistical analysis, we obtained all three samples (maternal, milk and infant samples together) from 72 patients, maternal and milk samples from 13 patients, and maternal and infant samples from 6 patients. 

Total serum and milk concentrations of VPA were measured by gas chromatography using a gas chromatograph (Chrom 5, Prague, Czech Republic) with a glass-packed column 1200 × 3 mm filled with 10% SP-1000 on 80/100 Supelcoport (Supelco, Bellefonta, PA, USA). Analysis was provided from milk samples without first defatting. To caprylic acid (the internal standard) in Eppendorf vials, 50 μL of serum or milk, 50 μL acetone and a small amount (approximately 30 mg) of solid ammonium sulfate were added and mixed. After centrifugation, 2 μL of the acetone layer was injected directly into the column for analysis using flame ionization detection. The performance characteristics of the method were as follows: linearity was between 5 and 125 mg/L, both for blood and milk. The accuracy and precision were validated by U.S. FDA rules; the within-day and between-day precision and accuracy were studied at three concentration levels in both matrices. At the tested concentrations, recovery in blood was between 97.2 and 103.5%, the coefficient of variation was 3.5–5.5%, recovery in milk was 90.8–99.1%, and the coefficient of variation was 3.4–6.1%. The limit of quantification was estimated as 1.0 mg/L. The method was quality controlled with external quality control RfB (Bonn, Germany) twice a year [14]. 

For statistical calculations, half of the lower limit of quantification (LLoQ) concentration was used for samples with concentrations lower than LLoQ [17,18]. The maternal apparent oral clearance (Cl) was calculated for VPA as follows [19]: Cl (L/kg) = daily dose (mg/kg)/maternal serum VPA concentration (mg/L) to evaluate the effect of the combination with ASM enhancing the elimination of VPA. Paired maternal serum, milk and infant serum concentrations were used to evaluate the milk/maternal serum concentration ratio and the infant/maternal serum concentration ratio. Paired concentrations of umbilical cord and infant serum were used to compare delivery with the colostrum period, and paired infant serum, colostrum, and mature milk concentrations were used to compare the colostrum and mature milk periods. We also evaluated the relationship between VPA concentrations in maternal serum, milk, and serum of breastfed infants. 

Statistical analysis was performed using GraphPad Prism version 5.00 for Windows and GraphPad Software (San Diego, CA, USA). The D′Agostino and Pearson omnibus normality test was applied to test whether the values came from a Gaussian distribution. Thereafter, we used the unpaired t-test (when the values follow the Gaussian distribution) or the nonparametric Mann–Whitney test for the comparison of the distributions of two unmatched groups, the paired t-test (the Gaussian distribution) or the nonparametric Wilcoxon signed-rank test for the comparison of the distributions of two matched groups, and the Pearson correlation test (the Gaussian distribution) or the Spearman nonparametric correlation test for the correlation analysis. A value of *p* < 0.05 was considered statistically significant.

## 3. Results

The basic characteristics of the mothers and their infants are summarized in Table 1.

**Figure 1:**
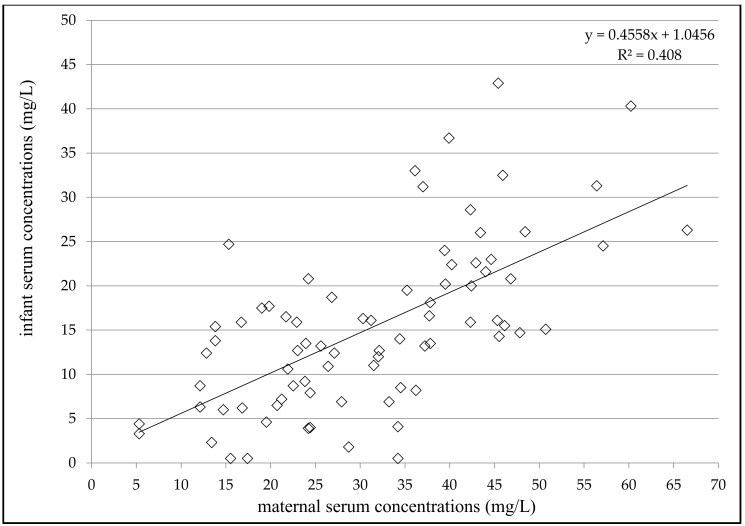
Correlation between infant and maternal serum concentrations of valproic acid 2–5 days after delivery; number = 78; *p* < 0.0001; Spearman correlation coefficient = 0.6338; 95% confidence interval = 0.4736–0.7534.

**Figure 2:**
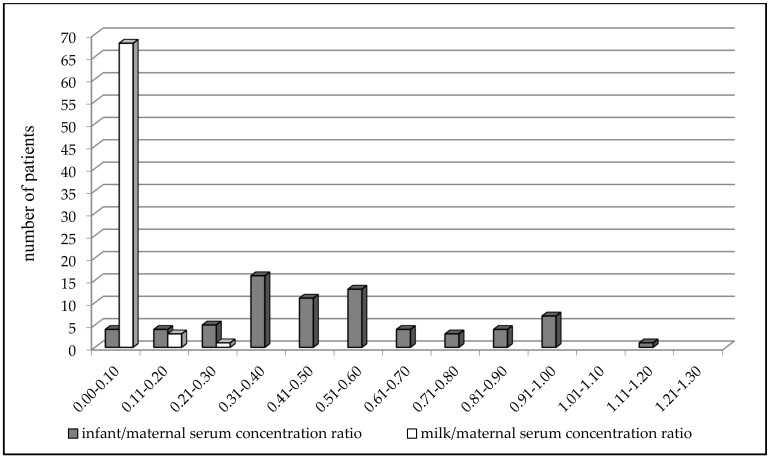
Comparison of the paired milk/maternal serum concentration ratio (*N* = 72, median = 0.03) versus the infant/maternal serum concentration ratio (*N* = 72, median = 0.47) of valproic acid 2-5 days after delivery; *p* < 0.0001, Wilcoxon signed-rank test.

VPA concentrations ranged from 4.3 to 66.5 mg/L (mean 31.2 ± 13.6 mg/L) in the maternal serum, from 0.5 to 5.9 mg/L (mean 1.1 ± 1.2 mg/L) in the milk, and from 0.5 to 42.9 mg/L (mean 15.4 ± 9.4 mg/L) in the infant serum (Table 2). Significant correlations were found between milk and maternal serum concentrations (*N* = 84, *p* = 0.0085, Spearman correlation coefficient = 0.2852), infant serum and milk concentrations (*N* = 72, *p* = 0.016, Spearman correlation coefficient = 0.2831) and infant and maternal serum concentrations (*N* = 78, *p* < 0.0001, Spearman correlation coefficient = 0.6338, Figure 1). Significant correlations were also observed between infant serum concentration and both the maternal daily dose of VPA (*N* = 70, *p* < 0.0001, Spearman correlation coefficient = 0.5045) and the dose related to maternal body weight (*N* = 62, *p* < 0.0001, Pearson correlation coefficient = 0.4887). The milk/maternal serum concentration ratio ranged between 0.01 and 0.22 (mean 0.04 ± 0.04), and the infant/maternal serum concentration ratio ranged from 0.01 to 1.61 (mean 0.51 ± 0.28) (Table 2). A comparison of the paired milk/maternal serum concentration ratio (*N* = 72, median = 0.03) and the infant/maternal serum concentration ratio (*N* = 72, median = 0.47) is shown in Figure 2 (*p* < 0.0001, Wilcoxon signed-rank test). 

Concomitant treatment with enzyme-inducing antiseizure medication (carbamazepine, phenytoin, phenobarbital, and primidone), but not with lamotrigine or topiramate, significantly increased the apparent maternal clearance of VPA (*p* = 0.0329) (Table 2). Only six (7%) of the maternal serum concentrations were measured in a reference range of 50–100 mg/L [16], and 93% were lower (none below the LLoQ). Concentrations in milk and infant serum did not reach the lower limit of the reference range used for the general epileptic population; 73% of milk and 4% of infant serum concentrations were below the LLoQ. VPA monotherapy was prescribed to 60% of women, 34% used a dual combination, and 6% received a triple combination with other ASMs. A comparison of paired VPA concentrations and their ratios during delivery versus the colostrum period and colostrum versus mature milk periods is shown in Table 3. 

**Figure 3:**
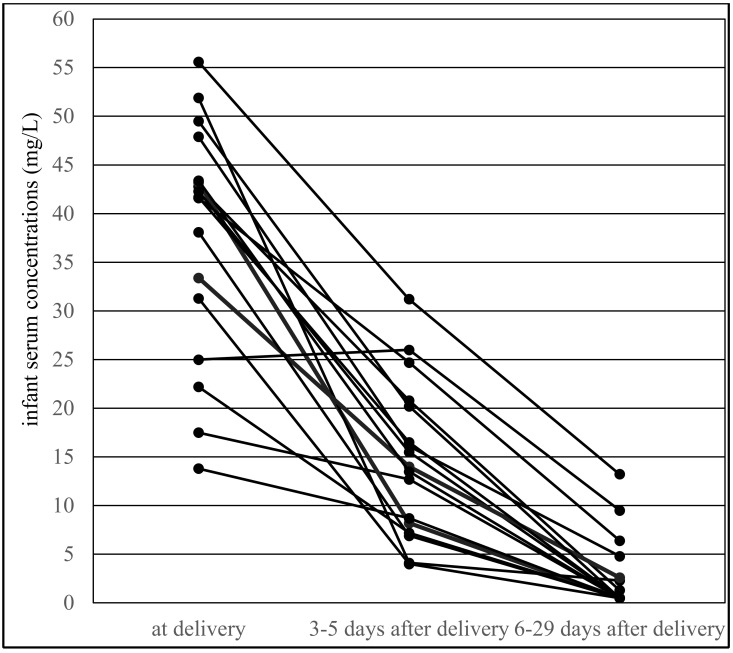
Valproic acid serum concentrations obtained from 17 breastfed infants at all three time points: at delivery, during the colostrum period (3–5 days after delivery, median 3 days) and in the mature milk period (6–29 days after delivery, median 10 days).

Paired infant serum concentrations were significantly lower than umbilical cord serum concentrations, and the paired infant/maternal serum concentration ratio was also significantly lower than the umbilical cord/maternal serum concentration ratio [14]. In contrast, both paired infant serum concentrations and paired infant/maternal serum concentration ratios were significantly higher during the colostrum period than during the mature milk period. The paired concentrations of colostrum and mature milk did not differ significantly between the two periods, similar to the paired colostrum/maternal serum and milk/maternal serum concentration ratio [15]. Serum VPA concentrations obtained from 17 breastfed infants at all three time points (at delivery, during the colostrum period and during the mature milk period) are shown in Figure 3.

## 4. Discussion

Although the prescription of VPA to girls/women has decreased significantly in the past decade [6,7,8,9,10], older ASMs, including VPA, are still widely used, especially in low- and middle-income countries [11,12,13]. For this reason, we hope that new data obtained from our study with the highest number of simultaneously analyzed maternal serum, milk and infant serum concentrations (from one center using a consistent methodology) compared to all previous studies using varying and incomparable criteria [17,20,21,22,23,24,25,26,27,28,29,30,31,32,33,34]. Table 4 will be useful for managing pregnant and breastfeeding epileptic women using VPA. 

Moreover, information related to the comparison of VPA concentrations in colostrum and mature milk, particularly in breastfed neonates versus both umbilical cord and breastfed infants during the mature milk period, is not available to our knowledge. In the available previous studies, VPA concentrations were measured simultaneously in mothers, milk and breastfed infants during the first 3–6 postnatal days only in a study from von Unruh et al. [20] with five cases (the infant/maternal serum concentration ratio ranged from < 0.04 to 0.40), and during the first postnatal week in a study from Meyer et al. [26] in three cases (average of the infant/maternal serum concentration ratio 0.62, range 0.25–1.05). The milk/maternal serum concentration ratio we observed was comparable with these two previously published studies [20,26].

A significant correlation was found between infant serum concentrations and milk concentrations, maternal serum concentrations, maternal VPA daily dose and the dose related to maternal body weight. However, only the dependence of the infant’s serum concentration on the maternal concentration, which may indicate the infant’s VPA exposure in the early postpartum period, may be clinically significant. Similarly, a highly significant correlation was observed between maternal and umbilical cord serum concentrations of VPA during delivery [14], but no correlation was demonstrated between maternal serum and milk concentrations or maternal and infant serum concentrations in the mature milk period [15]. In the present study, neither milk nor infant serum concentrations reached the lower limit of the reference range used for the general epileptic population, and 73% of the milk and 4% of the infant serum concentrations were below the LLoQ.

The paired milk/maternal serum concentration ratio of VPA was found to be much lower than the infant/maternal serum concentration ratio, which is supported by comparing the distribution of these two ratios in Figure 2, in which the infant/maternal serum concentration ratio shifted to higher values (i.e., “to the right”). This result was diametrically different from levetiracetam and topiramate, in which we observed that the mean milk/maternal serum concentration ratio was markedly higher than the mean infant/maternal serum concentration ratio, and that a movement of the infant/maternal serum concentration ratio to lower values (i.e., “to the left”) was apparent [35,36]. Paired colostrum and mature milk concentrations of VPA were measured on or under the lower limit of quantification, respectively, and were not significantly different between these two time periods. Conversely, paired infant serum concentrations obtained during the colostrum period were found to be significantly higher in comparison with the mature milk period and lower than the umbilical cord serum concentrations [14,15]. This result corresponds to Figure 3, which presents an almost continuous decrease in VPA-breastfed infant serum concentrations at three time points (delivery–colostrum–mature milk). Different declines in breastfed infant serum concentrations were observed for levetiracetam, in which a rapid decrease in concentrations was found between the delivery and colostrum periods and thereafter the concentrations were stable [35].

Milk production is a complex process involving transport mechanisms that are mainly mediated by two transporter superfamilies: ATP-binding cassette (ABC) and solute carrier (SLC). These proteins are localized in the basolateral or apical membrane of the mammary epithelium and participate in the uptake, reuptake or efflux of nutrients and compounds of different natures, including drugs. The presence of breast cancer resistance protein (BCRP/ABCG2) in the mammary gland has an important role in the transfer of nutrients, drugs and xenobiotics into milk. In contrast, the expression of peptide transporter 2 (PEPT2/SLC15A2) in the epithelial cells of the mammary gland could provide an efficient mechanism for the reuptake of short-chain peptide and peptide-based drug, and this mechanism may reduce the burden of xenobiotics in milk [37,38,39]. Although the role of transporters in the mammary gland is not fully elucidated and it is also unknown whether VPA is the substrate for any of these transporters, the effect of some reuptake transporters on negligible excretion of VPA in colostrum and mature milk is likely.

A combination with enzyme-inducing antiseizure medication (carbamazepine, phenytoin, phenobarbital, and primidone) significantly increased apparent maternal clearance of VPA, as in our previous study performed at delivery [14]. Therefore, the daily dose or the dose related to the mother’s body weight can be increased by approximately 30–40% to achieve VPA concentrations similar to those in women with VPA monotherapy. The effect of enzyme-inducing ASM was not observed in the following period of mature milk, probably due to the small number of patients in both subgroups [15].

From a clinical point of view, the exposure of breastfed infants to VPA is significantly lower than that during pregnancy. While the umbilical cord concentrations were approximately 1.5 times higher than the maternal concentrations, the serum concentrations of the breastfed infants reached only approximately half of the maternal value during the colostrum period and one tenth of the maternal value during the mature milk period. The minimal excretion of VPA in both colostrum and mature milk appears to be the reason for the rapid decline in infant concentrations. Therapeutic monitoring of serum VPA concentrations in breastfed infants is therefore not mandatory; however, if signs of possible adverse reactions are noted, infant serum concentrations should be determined.

There are some limitations of this study. The volume of breast milk ingested, the timing of feeding, and information about exclusive breastfeeding of the included infants are not known; however, this study presents a direct analysis of VPA concentrations in venous blood samples from breastfed infants along with measurements of drug concentrations in both mothers and milk. We were unable to demonstrate any relationship between VPA concentrations and clinical effects in either mothers or breastfed infants. No previous attempt has been made to correlate VPA concentrations with maternal seizure control during lactation. However, we hope that these new data from our study provide relevant information for the treatment of epilepsy during breastfeeding and the exposure of breastfed infants to VPA.

## 5. Conclusions

This study systematically analyzed VPA concentrations in breastfed mothers and the transport of VPA to colostrum and breastfed infants for more than 25 years in the largest group of patients ever reported to the best of our knowledge. Concomitant treatment with enzyme-inducing antiseizure medication (carbamazepine, phenytoin, phenobarbital, and primidone), but not lamotrigine or topiramate, significantly increased apparent maternal clearance of VPA during the first postnatal days. Concentrations in milk and infant serum did not reach the lower limit of the reference range used for the general epileptic population, and three-quarters of the concentrations in milk were lower than the lower limit of quantification. We have confirmed minimal VPA exposure in infants via colostrum or mature milk, and therefore routine monitoring of infant serum concentrations is not necessary. However, observation of breastfed infants is desirable, and if signs of potential adverse reactions are noted, infant serum concentrations should be measured.

## Figures and Tables

**Table 1:** Basic characteristics of the mothers and their infants.

		Number	Mean ± SD (Range)
Mothers:	age (years)	90	28 ± 5 (18–39)
concomitant antiseizure medication	carbamazepine (mg/day)	18	680 ± 291 (300–1200)
	lamotrigine (mg/day)	12	246 ± 108 (100–400)
	topiramate (mg/day)	4	200 ± 0 (200–200)
	phenytoin (mg/day)	3	230 ± 125 (100–350)
	levetiracetam (mg/day)	2	750 ± 354 (500–1000)
	primidone (mg/day)	1	500
	phenobarbital (mg/day)	1	55.5
Infants:	weight (kg)	75	3.2 ± 0.5 (2.0–4.3)
	length (cm)	68	49 ± 2 (43–53)
	female	41	
	male	37	

**Table 2:** Maternal apparent oral clearance (Cl), maternal (M), milk (Mi) and infant (I) serum concentrations of valproic acid (VPA) and their ratios in monotherapy (or in combination with levetiracetam) versus in combination with lamotrigine/topiramate (LTG/TPM) or enzyme-inducing antiseizure medication (carbamazepine, phenytoin, phenobarbital and primidone).

VPA Mono Mono	Weight (kg)	Dose (mg/day)	Dose (mg/kg)	M-Cl (L/kg)	M (mg/L)	Mi (mg/L)	I (mg/L)	Mi/M Ratio	I/M Ratio
number	48	49	43	43	55	54	48	54	48
median range	72 52–93	800 150–1250	11.3 1.7–19.2	0.34 0.08–1.04	31.2 4.3–66.5	0.5 0.5–5.9	15.5 0.5–42.9	0.03 0.01–0.22	0.52 0.01–1.00
mean ± SD	73 ± 11	717 ± 318	10.2 ± 4.6	0.37 ± 0.18	30.3 ± 13.7	1.2 ± 1.4	15.9 ± 9.7	0.04 ± 0.04	0.52 ± 0.25
VPA + LTG/TPM									
number	12	13	12	12	13	10	11	10	11
median range	66 52–82	900 300–1500	12.7 4.3–20.5	0.38 0.24–0.60	37.7 14.7–47.8	0.5 0.5–3.0	14.7 6.0–24.7	0.02 0.01–0.09	0.37 0.21–1.61
mean ± SD	67 ± 9	858 ± 385	13.2 ± 5.9	0.38 ± 0.12	33.7 ± 12.5	1.0 ± 0.9	13.8 ± 6.4	0.03 ± 0.03	0.49 ± 0.38
VPA + inducers									
number	18	18	14	14	22	20	19	20	19
median range	74 60–113	** 1000 300–2250	16.2 4.8–25.6	** 0.41 0.21–0.97	30.1 12.8–62.1	0.5 0.5–3.0	12.7 0.5–40.3	0.03 0.01–0.13	0.44 0.03–1.12
mean ± SD	76 ± 14	1064 ± 540	* 15.1 ± 7.1	0.47 ± 0.20	31.8 ± 14.3	1.0 ± 0.9	14.9 ± 10.5	0.03 ± 0.03	0.48 ± 0.30
Total									
number	78	80	69	69	90	84	78	84	78
median range	71 52–113	900 150–2250	12.2 1.7–25.6	0.36 0.08–1.04	31.8 4.3–66.5	0.5 0.5–5.9	14.5 0.5–42.9	0.03 0.01–0.22	0.48 0.01–1.61
mean ± SD	73 ± 12	818 ± 409	11.7 ± 5.7	0.39 ± 0.18	31.2 ± 13.6	1.1 ± 1.2	15.4 ± 9.4	0.04 ± 0.04	0.51 ± 0.28

* *p* < 0.005, ** *p* < 0.04; VPA, in monotherapy versus combination with enzyme-inducing antiseizure medication.

**Table 3:** Comparison of paired concentrations and ratios of valproic acid.

A	UC-d (mg/L)	I-c (mg/L)	UC/M-d Ratio	I/M-c Ratio				
number	41	41	41	41				
median range	41.6 10.7–72.1	* 15.1 0.5–42.9	1.40 0.64–2.49	* 0.47 0.03–1.61				
mean ± SD	37.6 ± 16.5	15.8 ± 9.1	1.48 ± 0.45	0.54 ± 0.30				
delivery × colostrum		* *p* < 0.0001		* *p* < 0.0001				
**B**	**I-c** **(mg/L)**	**I-m** **(mg/L)**	**Mi-c** **(mg/L)**	**Mi-m** **(mg/L)**	**I/M-c** **Ratio**	**I/M-m** **Ratio**	**Mi/M-c** **Ratio**	**Mi/M-m** **Ratio**
number	21	21	19	19	21	21	19	19
median range	14.0 0.5–26.1	2.3 0.5–9.5	0.5 0.5–5.9	0.5 0.5–3.0	0.49 0.03–1.61	* 0.09 0.01–0.22	0.03 0.01–0.13	0.02 0.01–0.09
mean ± SD	14.0 ± 8.1	* 3.1 ± 2.9	1.4 ± 1.5	0.9 ± 0.7	0.52 ± 0.34	0.09 ± 0.07	0.05 ± 0.04	0.03 ± 0.02
colostrum × milk		* *p* < 0.0001		not significant		* *p* < 0.0001		not significant

Delivery (d) versus colostrum period (c): comparison of paired umbilical cord (UC) versus breastfed infant (I) serum concentrations and paired umbilical cord/maternal (M) serum concentration ratio versus infant/maternal serum concentration ratio. Colostrum period (c) versus mature milk period (m): comparison of paired infant serum concentrations, milk (Mi) concentrations, infant/maternal serum concentration ratio and milk/maternal serum concentration ratio.

**Table 4:** Review of literature; Ref *=* reference, N_1_ *=* number of mothers, N_2_ *=* number of breastfed infants, M *=* maternal, Mi *=* milk, I *=* infant concentrations [17,20,21,22,23,24,25,26,27,28,29,30,31,32,33,34].

Ref.	N_1_	Postpartum Time	Maternal Dose	M (mg/L)	Mi (mg/L)	Mi/M Ratio	N_2_	I (mg/L)	I/M Ratio
[20]	16	4.3 ± 1.1 days range 3–6 days	22.1 ± 7.0 range 14.5–32.7 mg/kg/day	36.4 ± 14.0 range 18.6–66.5	1.8 ± 1.0 range 0.4–3.9	0.05 ± 0.03 range 0.01–0.10	5	< 1.0–13.4	< 0.04–0.40
[21]	13	up to 12th week	18.4 ± 7.2 mg/kg/day			0.025 ± 0.01			
[22]	6	4–19 weeks	750–1000 mg/day	39.4–79.0			6	0.7–1.5	0.01–0.02
[23]	6	3–82 days	9.5–31.0 mg/kg/day	4.7–102.2	0.034–5.4	0.027 ± 0.015 range 0.0071–0.052	2	0.50–0.55	
[24]	5			30.5–55.3	0.4–3.9	median 0.03 range 0.01–0.07	1 1	4.4 undetectable	
[25]	4	1st–3rd months	1200–1500 mg/day			0.05–0.10	1		0.08
[26]	4	1st week	1200–1800 mg/day	average 49.0 range 38.9–56.0	average 1.8 range 1.0–3.8	average 0.04 range 0.02–0.08	3	average 28.3 range 13.0–41.0	average 0.62 range 0.25–1.05
[17]	2	median 13 weeks range 5–20 weeks		30.0–41.9			2	7.5 i.e., <LLOQ	median 0.21 range 0.18–0.25
[27]	1 1	1.9 weeks 4.1 weeks	500 mg/day				1 1	< 0.0035 < 0.005	
[28]	1 1	6–17 days 1–43 days	1000 mg/day 1400 mg/day		1.4–3.0 1.4–3.5	0.02–0.03			
[29]	1 1	1 month 3 months	750 mg/day 500 mg/day	65.0 67.0			1 1	4.0 1.0	0.06 0.015
[30]	1	62 h 130 h	500 mg/day	9.9 34.3	0.18 0.46	0.02 0.01			
[31]	1	5 days 29 days	1600 mg/day		7.2 3.0	0.05–0.10	1	7.5 undetectable	
[32]	1	2 months	600 mg/day	14.9–34.3	< 0.4–2.0	< 0.02–0.06	1	< 0.4–2.0	
[33]	1	2nd week	2400 mg/day	100.0	7.0	0.07			
[34]	1	3 months	1200 mg/day				1	6.6	

## Data Availability

Authors declare that they take full responsibility for the data, the analyses and interpretation, and the conduct of the research; that they have full access to all of the data; and that they have the right to publish all data. Authors were not participating in industry-sponsored research and corporate activities for the evaluation of this manuscript.
